# Probiotic *Pediococcus pentosaceus* ABY 118 to Modulation of ChIFN-*γ* and ChIL-10 in Broilers Infected by *Eimeria tenella* Oocyst

**DOI:** 10.1155/2021/1473208

**Published:** 2021-10-08

**Authors:** Andreas Berny Yulianto, Lucia Tri Suwanti, Thomas Valentinus Widiyatno, Suwarno Suwarno, Muchammad Yunus, Wiwiek Tyasningsih, Sri Hidanah, Osfar Sjofjan, Widya Paramita Lokapirnasari

**Affiliations:** ^1^Sains Veteriner Doctoral Program, Faculty of Veterinary Medicine, Universitas Airlangga, Surabaya, Indonesia; ^2^Faculty of Veterinary Medicine, Wijaya Kusuma Surabaya University, Surabaya, Indonesia; ^3^Division of Veterinary Parasitology, Faculty of Veterinary Medicine, Universitas Airlangga, Surabaya, Indonesia; ^4^Division of Veterinary Pathology, Faculty of Veterinary Medicine, Universitas Airlangga, Surabaya, Indonesia; ^5^Division of Veterinary Microbiology, Faculty of Veterinary Medicine, Universitas Airlangga, Surabaya, Indonesia; ^6^Division of Animal Husbandry, Faculty of Veterinary Medicine, Universitas Airlangga, Surabaya, Indonesia; ^7^Faculty of Animal Husbandry, Brawijaya University, Malang, Indonesia

## Abstract

*Eimeria* causes coccidiosis, which has long been recognized as a disease in chickens that significantly affects the economy. The global chicken population continues to grow, and its contribution to food security increases, making it increasingly important to produce chicken meat that is safe for human and health. This study aims to prove *Pediococcus pentosaceus* ABY 118 to modulation of ChIFN-*γ* and ChIL-10 in chickens infected with *E*. *tenella* oocysts. This study used 100 of day-old chickens (DOC), randomly divided into 5 treatments; each treatment consists of 20 chickens. The treatments was as follows: *P*0 (−): negative control; *P*0 (+): positive control; *P*1: monensin; *P*2: probiotic 1.5 × 10^8^ CFU/ml; and *P*3: probiotic 3.0 × 10^8^ CFU/ml. At the age of 20 days, *Eimeria tenella* (*E*. *tenella*) oocysts were inoculated orally at a dose of 1 × 10^4^. The probiotic *P*. *pentosaceus* ABY 118 was given orally through drinking water from DOC to 35 days. Monensin was given orally through feed from the age of 14–26 days. The results of statistical analysis showed that there was a significant difference (*P* < 0.05) between treatments on ChIFN-*γ* and ChIL-10 at 6 and 8 days postinfected with *E*. *tenella* oocysts. Based on the results of this study, it can be concluded that the use of *P*. *pentosaceus* ABY 118 isolates at a dose of 1.5 × 10^8^ CFU/ml and 3.0 × 10^8^ CFU/ml per liter of drinking water can increase health by stimulation of ChIFN-*γ* and ChIL-10 in broiler infected with *E*. *tenella* oocyst.

## 1. Introduction

*Eimeria* is a genus of obligate intracellular parasites belonging to the phylum Apicomplexa; it is known that there are nine species of *Eimeria* in chickens, including *E*. *acervulina*, *E*. *brunetti*, *E*. *maxima*, *E*. *mitis*, *E*. *necatrix*, *E*. *praecox*, *E*. *tenella*, *E*. *mivati*, and *E*. *hagani*. Coccidiosis controlling in chickens in Indonesia is generally carried out through sanitation, administration of coccidiostats in feed and drinking water, and the use of coccidia vaccines [1]. In line with the WHO policy, to reduce excessive use of antibiotics in livestock and fisheries in accordance with Law Number 18, 2009, concerning livestock and animal health as already amended by Law Number 41, 2014, concerning amendments to Law Number 18, 2009, on livestock and animal health about the prohibition of the use of mixed feed certain hormones and/or feed additive antibiotics [2], it is necessary to develop alternative substance that can increase immunomodulation to coccidia.

The occurrence of anticoccidial drug resistance and the high cost of using coccidiosis vaccines in Indonesia are the underlying reasons, so it is necessary to find alternative methods as immunomodulators against coccidiosis. One alternative that can be pursued is to use probiotics. Some probiotics that are often used to improve performance in poultry are *Bifidobacterium*, *Lactobacillus casei*, and *L*. *acidophilus* [3–6]. The use of the probiotic *Pediococcus acidilactici* has been reported to provide protection against cases of coccidiosis caused by *E*. *acervulina* [7, 8]. *P*. *pentosaceus* belongs to the phylum Firmicutes, which can ferment undigested feed fiber into SCFA (short-chain fatty acids), especially butyrate [9], which has an influence on IEC (intestinal epithelial cells) in chickens, so that they can produce ChIL-18 which induces CD4+ cells to differentiate into Th1 effectors where ChIFN-*γ* produced by these cells will induce activated CD8+ to become CTLs when encountering antigens presented by *E*. *tenella* [10]. SCFA can also encourage CD4+ differentiation into Th1 memory. The proinflammatory response shown by CD4^+^ and CD8+ is also balanced by Tregs which are affected by SCFA produced by probiotic bacteria fermentation, so that ChIL-10 produced can relieve proinflammatory reactions. ChIL-10 is also produced by dendritic cells affected by SCFA from microbes [11, 12].

*P*. *pentosaceus* is known to modulate cellular immunity by upregulating the expression of cytokines interferon-gamma (IFN-*γ*) and tumor necrosis factor alpha (TNF-*α*) in the small intestine [13]. The combination of *P*. *acidilactici* and *Saccharomyces boulardii* probiotics is known to increase broiler resistance to coccidiosis by increasing humoral immunity [7, 8]. *Lactobacillus plantarum* (*L*. *plantarum*) can increase the number of CD4+ and CD8+ cells in the jejunum, lamina propria, and chicken tonsil ceca when compared to the use of antibiotics, where CD4+ showed a higher percentage increase. *L*. *plantarum* can also increase the expression of Th1 cytokines (IFN-*γ* and IL-12) and Th2 cytokines (IL-4) [14]. Cellular and humoral immune mechanisms are involved in the development of immunity against *Eimeria*, and gut-associated lymphoid tissue (GALT) in poultry is responsible for these mechanisms [15, 16]. Th1 produces proinflammatory cytokines (IFN-*γ*, TNF-*α*, and IL-2) which will trigger phagocytosis. Th2 produces cytokines including IL-4 which stimulates antibody production, IL-5 causes an eosinophil response to large extracellular parasites, but it also produces IL-6 and IL-13 cytokines [17]. Until now, the use of *P*. *pentosaceus* probiotics for enhancing immunity against coccidiosis has not been reported. The novelty of this research is that until now, the use of probiotics as immunomodulators of ChIFN-*γ* and ChIL-10 against coccidia is still very limited, so this study was conducted to prove that *P*. *pentosaceus* ABY 118 probiotics can be used as immunomodulators against *E*. *tenella* infection in broilers.

## 2. Materials and Methods

Kit ELISA : +Bio® (Georgia) Chicken IL-10 ELISA Kit, RayBio® (Georgia), Chicken IFN-gamma ELISA Kit, IFN-gamma microplate (96 wells (12 strips × 8 wells) coated with anti-chicken IFN-gamma, IL-10 microplate (96 wells (12 strips *x* 8 wells) coated with anti-chicken IL-10), wash buffer concentrate (20x), stop solution (0.2 M sulfuric acid), assay diluent *D*, assay diluent B, lyophilized standard, detection antibody IL-10 (biotinylated anti-chicken IL-10), detection antibody IFN-gamma (biotinylated anti-chicken IFN-gamma), streptavidin-conjugated HRP, TMB one-step substrate reagent (3,3,5,5′-tetramethylbenzidine (TMB) in buffer solution), distilled or deionized water, precision pipettes to deliver 2 *µ*l-1 *µ*l volumes, adjustable 1–25 *µ*l pipettes for reagent preparation, 100 *µ*l and 1 liter graduated cylinders, tubes to prepare standard and sample dilutions, absorbent paper, microplate reader capable of measuring absorbance at 450 nm, log-log graph paper or computer, and software for ELISA data analysis.

The research method was a completely randomized design (CRD) consisting of five treatments; each treatment has 20 chickens; the total sample used was 100 chickens from a hatchery. The treatments were as follows: *P*0 (−): negative control (noninfected); *P*0 (+): positive control (infected with *E*. *tenella* oocysts, without monensin, without probiotics); *P*1: infected with *E*. *tenella* oocysts, with monensin 0.15 g/kg feed; *P*2: infected with *E*. *tenella* oocysts, with probiotics 1.5 × 10^8^ CFU/ml; *P*3: infected with *E*. *tenella* oocysts, with probiotic 3.0 × 10^8^ CFU/ml. The treatment of giving probiotic started at the age of DOC. Monensin was given orally through feed from the age of 14 days to 26 days. The infection was carried out in 100 battery cage. At the age of 20 days, *E*. *tenella* oocysts were inoculated orally using a syringe with a dose of 1 × 10^4^.

### 2.1. Reagent Preparation

All reagents and samples were placed to room temperature (18–25°C) before use. Assay diluent *D* and assay diluent B should be diluted 5-fold with deionized or distilled water before use. Sample dilution: 1x assay diluent *D* should be used for dilution of serum. The suggested dilution for normal serum is 2-fold. Preparation of standard: briefly spin a vial of item C. Add 1000 *µ*l 1X assay diluent *D*; assay diluent *D* should be diluted 5-fold with deionized or distilled water before use) into item C vial to prepare a 10 ng/ml standard solution. Dissolve the powder thoroughly by a gentle mix. Pipette 300 *µ*l 1X assay diluent *D* into each tube. Use the 10 ng/ml standard solution to produce a dilution series. Mix each tube thoroughly before the next transfer. This procedure based on RayBio® Chicken IL-10 and Chicken IFN-gamma ELISA Kit.

### 2.2. Data Analysis

All data were analyzed using the analysis of variance (ANOVA) with *P* < 0.05. If the analysis result has a significant difference, it is followed with the Duncan multiple range test to observe the differences between treatments.

## 3. Results

### 3.1. ChIFN-*γ*

The results of statistical analysis showed that there was a significant difference (*P* < 0.05) between treatments on ChIFN-*γ* ELISA in the blood serum of chickens (6 days postinfection) infected with *E*. *tenella*. Treatment *P*1 showed a difference with all treatments, but *P*0 (negative) was not different from treatments *P*0 (positive), *P*2, and *P*3. The highest ChIFN-*γ* ELISA results in chickens (6 days postinfection) infected with *E*. *tenella* with *P*. *pentosaceus* ABY 118 were found in *P*1 treatment. The results of statistical analysis on ChIFN-*γ* - ELISA levels at 8 days postinfection showed that there was a significant difference (*P* < 0.05) between treatments. Treatment *P*0 (negative) showed no difference with *P*0 (positive) and *P*2, but *P*0 (negative), *P*0 (positive), and *P*2 showed a significant difference with treatment *P*1 and *P*3. The average results of ChIFN-*γ* ELISA in chickens (6 and 8 days postinfection) infected with *E*. *tenella* with *P*. *pentosaceus* ABY 118 are listed in Table 1.

### 3.2. ChIL-10

The results of statistical analysis showed that there was a significant difference (*P* < 0.05) between treatments on ChIL-10 ELISA in chickens infected with *E*. *tenella* on the 6^th^ postinfection day. Treatment *P*1 was significantly different from all treatments, and *P*1 showed the highest of ChIL-10. The average levels of ChIL-10 ELISA were equally low in treatments *P*0 (negative), *P*0 (positive), *P*2, and *P*3.

The results of statistical analysis of 8 days postinfection showed that there was a significant difference (*P* < 0.05) between treatments. ChIL-10 levels in treatment *P*0 (negative) showed no different results from treatment *P*0 (positive) and *P*2, but *P*0 (negative), *P*0 (positive), and *P*2 showed significant differences with treatments *P*1 and *P*3. Treatment *P*1 showed significant differences with all treatments. Treatment *P*2 showed no difference with *P*0 (negative), *P*0 (positive), and *P*3. The average results of ELISA ChIL-10 in chickens of 6 and 8 days after infection infected with *E*. *tenella* are listed in Table 2.

## 4. Discussion

### 4.1. ChIFN-*γ*

ChIFN-*γ* ELISA results in treatment *P*0 (positive) at 8 days postinfection showed the lowest number when compared to other treatments. According to [18, 19], interferon gamma (IFN-*γ*) is produced by *T* helper cells during *Eimeria* infection. This follows an increase in the number of CD4 and CD8 cells. Lilehoj [20] stated that IFN-*γ* has an inhibitory effect on *Eimeria*. The level of ChIFN-*γ* known by the ELISA test at the age of 6 days postinfection was 8.95 ng/ml, while at 8 days postinfection was 3.58 ng/ml. This decreased and low level of ChIFN-*γ* can occur because *Eimeria* suppresses the production of ChIFN-*γ* by stimulating Tregs to produce IL-10, so that these parasites can avoid cellular immune responses [21]. Interferon gamma that forms a microenvironment triggers a shift in the immune response that leads to cellular immunity regulated by Th1 cells and then activates cytotoxic T cells [22].

ChIFN-*γ* levels in treatment *P*1 showed the highest levels compared to other treatments. At 6 days post-*Eimeria* infection, ChIFN-*γ* levels were 26.20 ng/ml, and on day 8 postinfection, ChIFN-*γ* levels decreased to 17.06 ng/ml. In the treatment using monensin (*P*1), it was shown that monensin has an inhibitory effect on intracellular protein transport, so that cytokines accumulate in cells. The use of monensin causes the cytokine ChIFN-*γ*, which is produced by T cells, to be unable to leave the cell because it accumulates in the Golgi complex [23–26]. The inhibition of the release of ChIFN*-γ* from T cells will reduce the inhibitory effect on the intracellular development of *Eimeria*, so that oocysts which are the end results of the parasitic development process in the chicken indicate the highest results, eventhough the levels of ChIFN-*γ* detected in the ELISA assay showed the highest results among treatments (*P*0, *P*2, and *P*3). The 8 days postinfection showed a decrease in ChIFN-*γ* levels; this could occur because *Eimeria* stimulated Tregs to produce ChIL-10, which then shifted the dominance of Th1 towards Th2, so that the cellular immune response that was more suitable to overcome *Eimeria*'s intracellular infection was reduced in the effect, so that levels of ChIFN-*γ* decreased [21].

ChIFN-*γ* levels in *P*2 treatment, on day 6 postinfection, were lower than cytokine levels in *P*0 treatment, but the difference was on day 8 postinfection, where ChIFN-*γ* levels in *P*0 decreased, while ChIFN-*γ* levels in *P*2 increased. This illustrates the resistance to the development of *Eimeria* in the digestive tract of chickens, so that the ability of this parasite to suppress the production of ChIFN-*γ* by stimulating Tregs that produce ChIL-10 is suppressed.

*P*. *pentosaceus* belongs to the genus *Pediococcus*, family Lactobacillaceae, class Bacilli, phylum Firmicutes. Pediocci are lactic acid bacteria (LAB) and Gram-positive. Cell wall molecules are key ligands of probiotics that can interact with receptors and induce host signaling pathways. Most LAB, which are Gram-positive bacteria, have cell walls with a thick layer of peptidoglycan, containing various proteins, teichoic acids, and polysaccharides. The major macromolecules in the cell wall have essentially the same structure between species, but various modifications, such as in their glycosylation, can contribute to the strain-specific properties of probiotics [27, 28].

ChIFN-*γ* levels in the *P*3 treatment had the same pattern as the *P*2 treatment, namely, an increase in levels on the 6^th^ day postinfection and higher levels on the 8^th^ day postinfection. The difference is that the levels of ChIFN-*γ* in the *P*3 treatment were higher than in the *P*2 treatment. This is because more *P*. *pentosaceus* ABY 118 probiotics are available to activate APC cells, so that more T cells are activated, which in this case are Th1 cells, which then produce more ChIFN-*γ* than the *PP*2 treatment.

### 4.2. ChIL-10

ChIL-10 levels obtained from the ELISA test results on day 6 after *E*. *tenella* infection in the *P*0 treatment group were lower than in the *P*1 treatment group, but there was no difference with the treatment groups *P*2 and *P*3. ChIL-10 levels on the 8^th^ day postinfection in the *P*0 and *P*1 treatment groups decreased compared to the 6^th^ day postinfection, while the *P*2 and *P*3 treatment groups increased on the 8^th^ day postinfection.

The decreased ChIL-10 levels in the *P*0 treatment group are associated with ChIFN-*γ* levels; this is because *Eimeria* stimulates Tregs to produce IL-10, so that these parasites can avoid cellular immune responses [21, 29, 30]. The highest level of ChIL-10 in the *P*1 treatment group (8.68) was on day 6 postinfection and then decreased on day 8 (6.42) in principle not different from the results of ChIFN-*γ*. The high levels of ChIL-10 and ChIFN-*γ* were due to monensin given to the *P*1 treatment group having an inhibitory effect on protein transport, so that these cytokines accumulated in the Golgi complex. Monensin is known to be used in the detection of intracellular cytokines by flowcytometry. The number of stained activated T cells and the number of cytokine supernatants showed an inverse relationship [23, 26].

ChIL-10 levels in the *P*2 and *P*3 treatment groups showed a similar pattern to ChIFN-*γ* levels in the two treatment groups. The levels of these two types of cytokines were initially lower than *P*0 on day 6 postinfection and increased on day 8 postinfection. It is known that the effect of IL-10 as a key immunoregulator in viral, bacterial, fungal, protozoa, and helminth infections is to ameliorate the overresponse of Th1 cells and CD8+ T cells (which usually results in overproduction of IFN-*γ* and TNF-*γ*). IL-10 controls the immune response to limit the side-effects of host cells during inflammation [31, 32].

The increased ChIL-10 levels in the *P*2 and *P*3 treatment groups, given the probiotic *P*. *pentosaceus* ABY 118, could be associated with the production of microbial metabolites. These metabolites are in the form of short-chain fatty acids (SCFA), which are the results of bacterial fermentation [33]. The function of microbial metabolites as controllers of the development and differentiation of the immune system include SCFA, tryptophan metabolites, and retinoic acid [34, 35]. Bacterial fermentation products, especially SCFA, include acetate (C2), propionate (C3), and butyrate (C4). Acetate is the main fermentation product in chicken cecum, and chickens that received *P*. *pentosaceus* supplementation on average had a higher SCFA content. In the chicken cecum microflora, the number of bacteria from the phylum Bacteroidetes was positively correlated with the content of propionate, butyrate, and isobutyrate, while the increase in the acetate content was positively correlated with the number of bacteria from the phylum Firmicutes [9, 33].

The presence of proinflammatory ChIFN-*γ* cytokines balanced with anti-inflammatory ChIL-10 can create conditions of immune homeostasis. Every organism has a mechanism to maintain homeostasis in its body. The nervous system and endocrine system are known to be part of the mechanisms that regulate homeostasis. The immune system is a biophylactic system that protects an organism from attack by foreign organisms such as bacteria. Therefore, it means that the immune system does not only function in biophylaxis but also maintains homeostasis. This is because the immune system has the ability to respond to stimulation from the external environment [36]. ChIL-10 levels in the *P*2 treatment group which were higher than in the *P*3 treatment group could indicate that the levels of *P*. *pentosaceus* ABY118 isolate were associated with more SCFA which would also increase ChIL-10 levels.

## 5. Conclusion

Probiotic *P*. *pentosaceus* ABY 118 can be used to modulate the increase in ChINF-*γ* and ChIL-10 in broiler chickens infected with *E*. *tenella* oocysts at doses of 1.5 × 10^8^ CFU/ml and 3.0 × 10^8^ CFU/ml.

## Figures and Tables

**Table 1:** The average value of ChIFN-*γ* ELISA (ng/ml) in the blood serum of chickens (6 and 8 days postinfection) infected with *E*. *tenella*.

Treatment	The average value of ChIFN-*γ* ELISA (ng/ml) of chickens (6 days postinfection)	The average value of ChIFN-*γ* ELISA (ng/ml) of chickens (8 days postinfection)
*P*0 (negative)	10.45^a^ ± 7.63	6.86^a^ ± 3.22
*P*0 (positive)	8.95^a^ ± 4.45	3.58^a^ ± 1.88
*P*1	26.20^b^ ± 12.81	17.06^b^ ± 9.35
*P*2	3.74^a^ ± 1.49	7.15^a^ ± 1.00
*P*3	7.16^a^ ± 2.34	14.77^b^ ± 0.18

*Note*. ^a,b^Different superscripts in the same column indicate that there is a significant difference (*P* < 0.05) between treatments.

**Table 2:** The average value of ChIL-10 ELISA (ng/ml) in the blood serum of chickens (6 and 8 days postinfection) infected with *E*. *tenella*.

Treatment	The average value of ChIL-10 ELISA (ng/ml) of chickens (6 days postinfection)	The average value of ChIL-10 ELISA (ng/ml) of chickens (8 days postinfection)
*P*0 (negative)	1.98^a^ ± 0.82	1.37^a^ ± 1.11
*P*0 (positive)	2.07^a^ ± 1.20	0.83^a^ ± 0.31
*P*1	8.68^b^ ± 3.22	6.42^c^ ± 2.87
*P*2	0.98^a^ ± 0.93	2.49^ab^ ± 1.16
*P*3	1.08^a^ ± 0.53	3.80^b^ ± 3.31

*Note*. ^a,b^Different superscripts in the same column indicate that there is a significant difference (*P* < 0.05) between treatments.

## Data Availability

The data used in this study are available from the authors upon request.
